# Out of *Xanthopygus* (Coleoptera: Staphylinidae): escape from polyphyly

**DOI:** 10.3897/zookeys.1071.75947

**Published:** 2021-11-17

**Authors:** Stylianos Chatzimanolis

**Affiliations:** 1 Department of Biology, Geology and Environmental Science, University of Tennessee at Chattanooga, 615 McCallie Ave., Dept. 2653, Chattanooga, TN, USA University of Tennessee at Chattanooga Chattanooga United States of America

**Keywords:** New genus, phylogeny, Staphylininae, Staphylinini, Xanthopygina

## Abstract

*Xanthopygus* as currently defined is the largest genus in the subtribe Xanthopygina (Coleoptera: Staphylinidae: Staphylininae) with 40 described species. However, the genus is poorly defined, morphologically heterogeneous and previous studies have questioned whether it is a natural group. A morphological (51 characters) Bayesian phylogenetic analysis was performed to test whether *Xanthopygus* is a monophyletic group. The analysis indicated that *Xanthopygus* was polyphyletic, and therefore species were split into four different genera. *Xanthopygusnigricornis* Scheerpeltz was transferred to *Oligotergus* as *Oligotergusnigricornis***comb. nov.** and *Xanthopygusskalitzkyi* (Bernhauer) was transferred to *Styngetus* as *Styngetusskalitzkyi***comb. nov.** A new genus, *Photinopygus***gen. nov.** was erected to accommodate the majority of the species previously in *Xanthopygus* and *Xanthopygus* sensu novo is used in a new restricted sense to accommodate the remaining species. Diagnostic features are provided to distinguish species in the genera *Photinopygus* and *Xanthopygus* from each other and all other Xanthopygina genera.

## Introduction

Recently, Chatzimanolis and Brunke (2019) produced a comprehensive phylogeny of the subtribe Xanthopygina using morphological and molecular data and established several lineages within Xanthopygina. Catapulting from that work was the description of several new genera of Xanthopygina (Chatzimanolis 2019; Chatzimanolis and Hightower 2019; Chatzimanolis and Brunke 2021) that were placed in a phylogenetic framework. And while description of new genera is always exciting, many problems still exist with genera that have received little taxonomic attention over the last 200 years. One of the most problematic areas within Xanthopygina is the *Xanthopygus* lineage (briefly summarized in the next paragraphs), a group that includes *Elecatopselaphus* Scheerpeltz, *Phanolinus* Sharp, *Triacrus* Nordmann, *Xanthopygus* Kraatz, *Xenopygus* Bernhauer, along with *Gastrisusnitidus* Bernhauer and Genus 1 (a potentially undescribed new genus).

*Phanolinus* is perhaps one of the most charismatic taxa within Xanthopygina, and even Staphylinidae, with bright metallic coloration covering the whole body. However, many species were described solely based on the differences in coloration and many of them are potential synonyms (Chatzimanolis unpublished data). *Elecatopselaphus* was recovered as the sister group of *Phanolinus* (Chatzimanolis and Brunke 2019) and whether or not it should be treated as a separate genus or *Phanolinus* is still a matter of investigation. *Xenopygus* was revised by Caron et al. (2016) and Chatzimanolis and Caron (2016) provided clarifications and additions, but it is doubtful that the two species groups currently recognized in *Xenopygus* form a monophyletic group (Chatzimanolis and Brunke 2019; and this paper). *Gastrisusnitidus* and Genus 1 may belong to the same (new) genus, but more data and analyses are needed to clarify their position. *Triacrus* was shown to be nested within *Xanthopygus* (Chatzimanolis & Brunke 2019) but without support.

**Figure 1.**
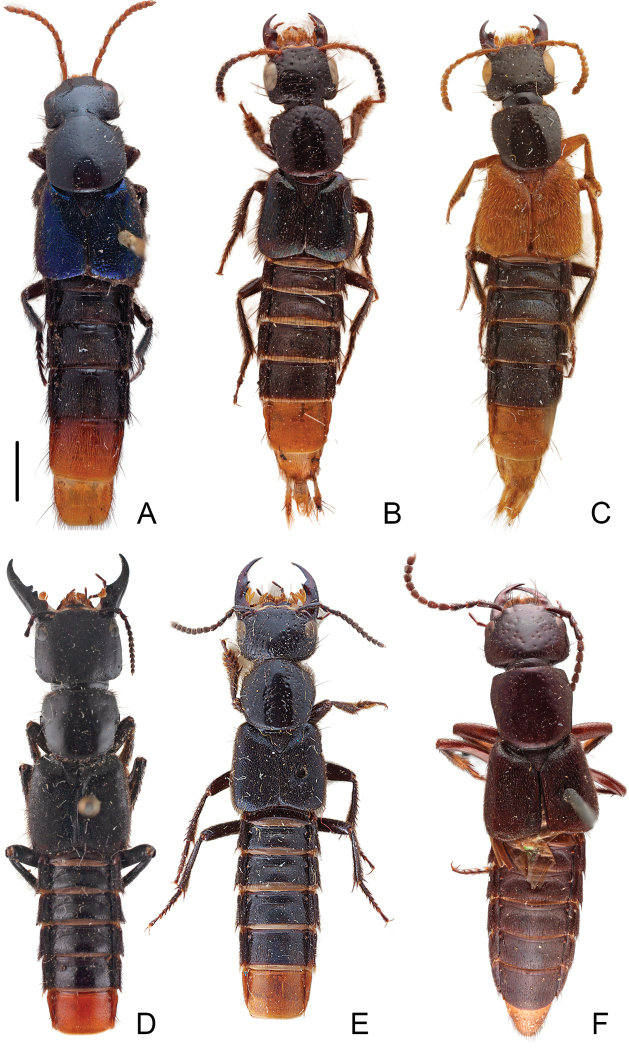
Habitus photographs of species of *Xanthopygus* sensu Herman 2001**A***Xanthopyguscalidus* (Er.) **B***Xanthopyguschapareanus* Scheerpeltz **C***Xanthopygusdimidiatus* Bernhauer. Species **A–C** are transferred to *Photinopygus* gen. nov. **D***Xanthopygusgiganteus* (Bernhauer) **E***Xanthopygusxanthopygus* (Nordmann) **F***Xanthopygusskalitzkyi* (Bernhauer), transferred to *Styngetus*. Scale bars: 1.8 mm (**A**); 1.7 mm (**B**) 1.8 mm (**C**); 3.8 mm (**D**); 3.0 mm (**E**); 2.0 mm (**F**).

*Xanthopygus* (Fig. 1) as currently defined (referred to as *Xanthopygus* sensu Herman to include all taxa of *Xanthopygus* as presented in Herman’s 2001 catalogue) is the most speciose genus in Xanthopygina with 40 valid species. The name *Xanthopygus* refers to the bright yellow or orange coloration of segments 7 and 8. Notes on the biology of adults and larvae are known for *Xa.cognatus* Sharp (Quezada et al. 1969) but the biology of the remaining species of the genus is largely unknown. Since there is no comprehensive taxonomic treatment of *Xanthopygus*, there are no good characters to define the genus, which has been typically diagnosed with a combination of the following: superior marginal line of pronotal hypomeron not continuing to anterior margin, postcoxal process present, and tergites 3–5 with arch-like carina (e.g., Hayashi 1997; Navarrete-Heredia et al. 2002; Navarrete-Heredia 2004; Rodríguez et al. 2012). Unfortunately, these three character states are not unique for *Xanthopygus* and have arisen multiple times within the subtribe (Chatzimanolis and Brunke 2019; and this paper), and as early as 2014; Chatzimanolis (2014) hypothesized that *Xanthopygus* is not monophyletic.

*Xanthopygus* was described by Kraatz (1857) and included the species that Erichson (1839; 1840) listed in ‘*Staphylinus* Fam. VII and *Philonthus* Erichs. pro parte’ (Herman 2001). Species in Fam. VII included (in the order listed by Erichson) *Staphylinussapphirinus* Er., *St.calidus* Er., *St.hilaris* Er., *St.tepidus* Er., *St.iopterus* Er., *St.cyanelytrius* Er., *St.chrysopygus* Er., *St.pyraster* Er., (a junior synonym of *St.haemorrhoidalis* Germar also listed by Erichson), *St.chrysurus*, and *St.faustus* Er. Kraatz (1857, p. 540) listed the species *Philonthusxanthopygus* (Nordmann), *Ph.herilis* Er., *Ph.analis* Er., *Ph.bicolor* (Laporte), and *Ph.mirabilis* Er. as those he intended to move from ‘*Philonthus* Erichs. pro parte’ to *Xanthopygus*. As stated by Herman (2001), all included species in a group must be cited by available names according to Article 67.2.1 of the ICZN (ICZN, 1999). Thus, the first included species in *Xanthopygus* were those cited by Gemminger and Harold (1868) who included in *Xanthopygus* all the species listed above and used *X.abdominalis* Gemminger and Harold as a replacement name (without justification) for *X.Xanthopygus* (Nordmann). *Xanthopygusabdominalis* has been treated as junior synonym of *X.xanthopygus* (Nordmann) by all subsequent authors. Sharp (1876) added several more species to *Xanthopygus* and was the first one to recognize that the genus (as proposed by Kraatz) was morphologically heterogeneous. Later, Sharp (1884) established the genus *Lampropygus* Sharp to include *L.xanthopygus* (Nordmann), *L.cognatus* (Sharp), *L.analis* (Er.) and *L.bicolor* (Er.). Unfortunately, the characters (ligula less emarginate, pronotum anterolaterally restricted) provided by Sharp (1884) to establish the concept of *Lampropygus* are present in *Xanthopygus* as well. In 1906, Bernhauer (1906) placed the last two species (*analis* and *bicolor*) in the genus *Xenopygus* Bernhauer. Based on his publications (e.g., Bernhauer 1905, 1906, 1917, 1927), Bernhauer agreed with Sharp on the concepts of *Xanthopygus* and *Lampropygus* as established by Sharp, although neither Sharp nor Bernhauer provided clear diagnostic characters for these genera. Bernhauer (1906) established the subgenus Heteropygus Bernhauer for two particularly large species, *L.giganteus* Bernhauer and *L.oliveirae* (Lynch) in *Lampropygus*. Lucas (1920) designated *L.xanthopygus* as the type species of *Lampropygus*. Blackwelder (1943) seemingly ignored the generic concepts that had been established by Sharp and Bernhauer for *Lampropygus* and *Xanthopygus*, and designated *L.xanthopygus* (Nordmann) as the type species of *Xanthopygus*, which resulted in *Lampropygus* becoming a junior synonym of *Xanthopygus*. This nomenclatural act established the concept of *Xanthopygus* as it stands today before the results of this paper. Perhaps to his credit, Blackwelder (1943; p.450 footnote) realized that he was giving a new meaning to *Xanthopygus* and suggested that new generic assignments would be needed in the future for some of the species in *Xanthopygus*.

While it is rather obvious from the taxonomic history above that *Xanthopygus* is not homogeneous, the goal of this paper is to use a phylogenetic framework to show that *Xanthopygus* sensu Herman can be confidently split into two or more taxa. Additionally, I seek to define diagnostic characters that can easily separate the various groups within *Xanthopygus*.

## Materials and methods

### Taxon sampling

The focus of this paper was to determine whether the species currently in *Xanthopygus* sensu Herman form a monophyletic group. Thus, the analysis conducted focused on this goal rather than attempting to decipher the exact placement of all the different *Xanthopygus* species groups within Xanthopygina. For the ingroup, I included 21 species from *Xanthopygus*, comprising all the different species groups in that genus (Chatzimanolis unpublished data). *Xanthopygusborealis* Hatch was listed as a valid species of *Xanthopygus* by Herman (2001) but that species is a junior synonym of *Tympanophoruspuncticollis* (Erichson). I also included 14 species as outgroup taxa, which included representatives of all genera belonging in the *Xanthopygus* lineage except *Elecatopselaphus*. From the *Xanthopygus* lineage I included the following taxa: *Gastrisusnitidus*, an undescribed taxon referred to as Genus 1 (Chatzimanolis and Brunke 2019), *Phanolinuscolombinus* Bernhauer, *Triacrusdilatus* Nordmann, and four species of *Xenopygus*, representing both species groups within *Xenopygus*. In addition to the taxa of the *Xanthopygus* lineage, I included species from *Gastrisus* Sharp, *Oligotergus* Bierig and *Styngetus* Sharp since the overall habitus of these taxa is sometimes confused with that of *Xanthopygus*, and *Philothalpus* Kraatz (as distant outgroup). I examined the type specimens of all ingroup taxa included in the analyses, except for *Xa.cyanelytrius* (Perty), *Xa.oliveirae* Lynch and *Xa.pexus* (Motschulsky) that are considered lost. Specimens were examined from the following collections: the American Museum of Natural History (AMNH), the Natural History Museum of London (BMNH); the Canadian National Collection of Insects, Arachnids and Nematodes (CNC), the Field Museum (FMNH); the Naturhistorisches Museum Wien (NMW); the Natural History Museum of Denmark (NHMD), the Senckenberg Deutsches Entomologisches Institut (SDEI), the Snow Entomological Collection, Biodiversity Institute, University of Kansas (SEMC), the University of Tennessee at Chattanooga Insect Collection (UTCI), and the Museum für Naturkunde der Humboldt-Universität (ZMHB). A DarwinCore format file with the voucher numbers for all the material examined can be found as Suppl. material 1. Because not all specimens had catalogue numbers, I added a new label to every specimen examined to serve as the voucher number; these labels had the following format: ‘*Xanthopygus* phylogeny voucher SC-123’. In addition to the specimens listed in the Supp. File 1, I have access to virtually all specimens of *Xanthopygus* sensu Herman since I have borrowed materials from museums around the world for the revisions, and I had the ability to check a wide range of specimens for characters that are difficult to observe.

### Specimen preparation

Specimens were examined using an Olympus ZX10 stereomicroscope either as dry mounts or disarticulated in glycerin. Photographs of species were taken using a Canon 40D camera equipped with a MP-E 65 mm macro lens on a Cognisys StackShot 3X macro rail and controller (https://cognisys-inc.com/stackshot-macro-rail-package.html). Images were automontaged using Helicon Focus Pro v.7.7.4 (http://www.heliconsoft.com/heliconsoft-products/helicon-focus/) and post-processed in Adobe Photoshop v.22.3. Tree diagrams were first processed using FigTree v.1.4.4 (http://tree.bio.ed.ac.uk/software/figtree/) and then edited in Adobe Illustrator v.25.2.

### List of morphological characters

In total, 51 morphological characters were scored in Mesquite v.3.61 (Maddison and Maddison 2019). Some characters were derived from Chani-Posse et al. (2018) or Chatzimanolis and Brunke (2019) but several are novel for Staphylinini phylogenetics. All characters were treated as unordered and neither invariant nor autapomorphic characters were included in the analyses. Figures from this manuscript are cited as Fig. #; figures from other citations are cited as follows: Fig. CA#: Chatzimanolis and Ashe (2005); Fig. CB#: Chatzimanolis and Brunke (2019); Fig. CC#: Chatzimanolis and Caron (2016); Fig. CH#: Chatzimanolis (2017); Fig. CP#: Chani-Posse et al. (2018); Fig. CT#: Chatzimanolis (2015a); Fig. L#: Li and Zhou (2011); Fig. S#: Smetana and Davis (2000). An * denotes novel character for Staphylinini phylogenetics.

1*. Antennae, antennomere 1 in comparison to antennomere 2: (0) less than twice as long; (1) twice as long or longer.

**Figure 2.**
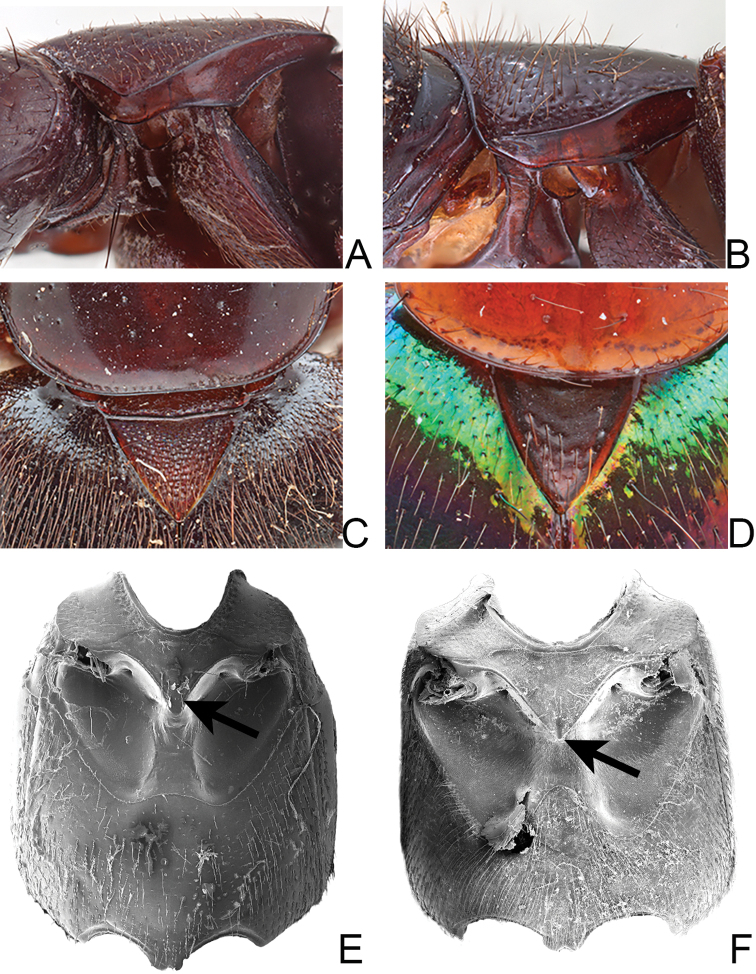
Diagnostic characters for *Xanthopygus***A** pronotal hypomeron of *Xanthopygusskalitzkyi* (Bernhauer) **B** pronotal hypomeron of *Xanthopygusxanthopygus* (Nordmann) **C** mesoscutellum of *Xanthopyguscognatus* Sharp **D** mesoscutellum of *Xanthopygusmirabilis* (Erichson) **E** mesoventrite of *Xanthopygusmirabilis* (Erichson), arrow points to intercoxal process **F** mesoventrite of *Xanthopygusxanthopygus* (Nordmann), arrow points to intercoxal process. Not to scale.

2. Antennae, antennomere 4, tomentose pubescence: (0) absent (Fig. 1D); (1) present (Fig. 1A–C, E, F).

3. Antennae, antennomere 4: (0) elongate (Fig. 1A, F); (1) subquadrate (Fig. 1D).

4. Antennae, antennomere 5: (0) elongate (Fig. 1A, F); (1) subquadrate (Fig. 1D, E); (2) transverse (Fig. CT2).

5. Antennae, antennomere 6: (0) elongate (Fig. 1F); (1) subquadrate (Fig. 1C); (2) transverse (Fig. CT2).

6. Antennae, antennomere 7: (0) elongate (Fig. 1F); (1) subquadrate (Fig. 1A); (2) transverse (Fig. CT2).

7. Head, length in comparison to pronotum: (0) shorter (Fig. 1A–C, E, F); (1) subequal (Fig. 1D).

8. Head, width in comparison to pronotum: (0) narrower (Fig. CC1–2); (1) subequal (Fig. 1A–C, F); (2) wider (Fig. 1D–E).

9*. Head, shape, posterior margin: (0) slightly extended posteriad on each side of the neck (Fig. 1A–C); (1) more or less at same level with neck border (Fig. 1D–F).

**Figure 3.**
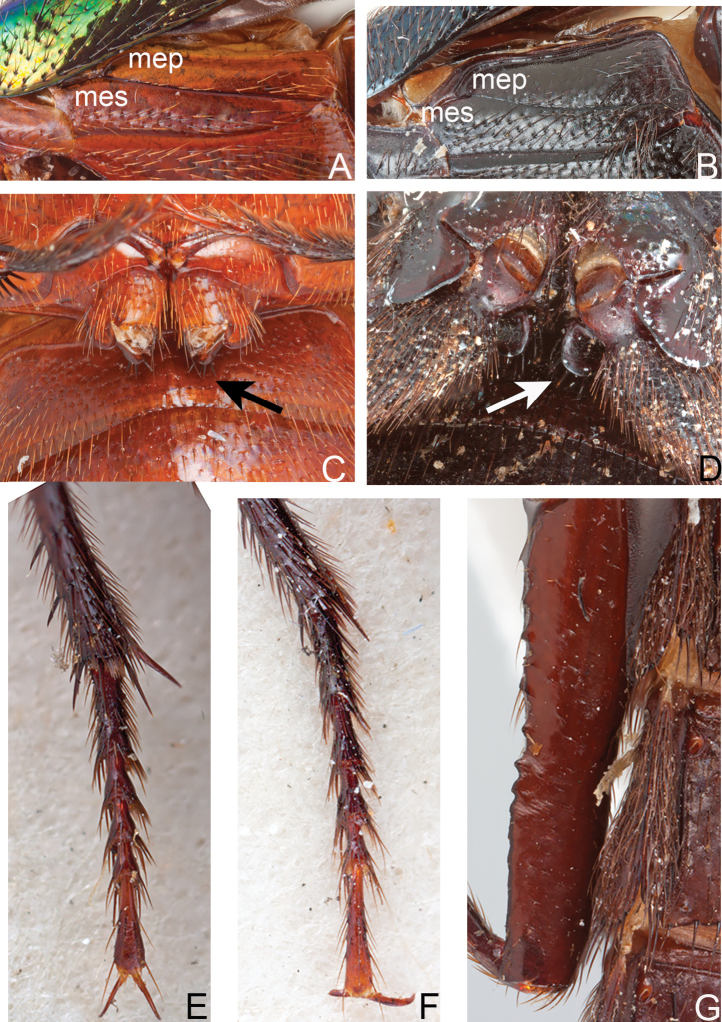
Diagnostic characters for *Xanthopygus***A** metepimeron (mep) and metepisternum (mes) of *Xanthopygusmirabilis* (Erichson) **B** metepimeron (mep) and metepisternum (mes) of *Xenopygusanalis* (Erichson) **C** metacoxae of *Xanthopygusmirabilis* (Erichson), arrow points to spines **D** metacoxae of *Triacrusdilatus* Nordmann, arrow points to spines **E** Metatarsus of *Xanthopygusxanthopygus* (Nordmann) **F** metatarsus of *Xanthopygusflohri* Sharp **G** metafemur of *Xanthopygusskalitzkyi* (Bernhauer), showing crenulate surface. Not to scale.

10. Head, eye size relative to length of head (length of head measured from anterior margin of clypaeus to posterior margin of head): (0) small (less than 2/5 length of head) (Fig. 1D); (1) medium (between 2/5 and 2/3 length of head) (Fig. 1B); (2) large (more than 2/3 length of head) (Fig. CC1–2).

11. Labrum, emargination, shape: (0) V-shaped, lobes moderately separated; (1) broadly U-shaped, lobes strongly separated (Fig. 1B–C); (2) narrow, lobes separated by a thin channel.

12. Head, deep punctures demarcating raised postmandibular ridge dorsolaterally: (0) absent (Fig. CT3); (1) present (Fig. S81).

13. Hypostomal cavity (hc): (0) hc moderately delimited (i.e., cavity surface without microsculpture or punctation different from rest of nearby head surface) (Figs S8, S10); (1) hc slightly delimited (cavity distinct only laterally, its surface with same microsculpture or punctuation as rest of nearby head surface).

14. Mandible, curvature: (0) more or less straight, except tip of mandible (Fig. 1D); (1) curved from apical (distal) half (Fig. 1B–C, E–F).

15. Mandible, left, teeth structure (excludes tip of mandible): (0) one tooth (Fig. CA14); (1) two teeth, separated by deep emargination (Fig. 1D); (2) one bicuspid tooth (Fig. 1C); (3) one tooth and one bicuspid tooth (in the same proximodistal succession; Fig. CT2).

16. Neck, disc (i.e., dorsal surface of neck not including dorsolateral areas): (0) punctures absent or rather sparse (Fig. 1A, C–D, F); (1) with dense, moderately coarse punctures (Figs CC1–2).

17*. Pronotum, microsculpture: (0) polygon shaped; (1) with transverse lines (seen easily at 70× magnification); (2) with dense micropunctures (Figs CA30, 32,38); (3) with sparse micropunctures (but no transverse lines visible at 70× magnification).

18. Prothorax, disc of pronotum, distribution of punctures: (0) median part of pronotum with punctation beyond midlength (Fig. 1); (1) median part of pronotum with punctation not continuing beyond midlength (Fig. SB2: *Gastrisus*).

19. Prothorax, disc of pronotum, distribution of punctures if punctures continue beyond midlength: (0) more or less homogeneous (i.e., punctures are separated by same distance; Fig. 1A); (1) with large impunctate areas between punctures (i.e., punctures not equally distributed; Fig. 1B–C).

20. Prothorax, hypomeron, inferior marginal line (iml), development: (0) iml not continued as a separate entity beyond anterior pronotal angles (Fig. S42–44); (1) iml continued as a separate entity beyond anterior pronotal angles and curving around them (Fig. S53).

21. Prothorax, hypomeron, superior marginal line: (0) continuous to anterior margin (Fig. 2A); (1) not continuous to anterior margin (Fig. 2B).

22*. Prothorax, hypomeron, angles of superior and inferior marginal lines: (0) superior and inferior line produce anterolateral angles parallel to one other (Fig. 2A); (1) superior and inferior line produce anterolateral angles not parallel to one other (Fig. 2B).

23. Prothorax, postcoxal process: (0) absent; (1) present (Fig. S53).

24. Prothorax, basisternum (bs), length relative to length of furcasternum (fs) (bs/fs, measured laterally): (0) bs slightly to moderately longer than fs (bs/fs ratio up to 1.5); (1) bs distinctly longer than fs (bs/fs ratio >> 1.5) (Fig. CP8A).

25. Prothorax, basisternum, position of pair of macrosetae (ms, if present) in relation to anterior margin of prosternum (amp) and the sternacostal suture (ss): (0) ms situated close to amp (i.e., not farther than one fourth the distance between amp and the ss along midline) (Fig. S86); (1) ms situated far from amp (i.e., farther than one fourth the distance between amp and the ss along midline) (Fig. L11A, B, E, F).

26. Mesothorax, elytra, with contiguous polygon-shaped meshed microsculpture (elytra appearing matt): (0) absent; (1) present (Fig. SB2: *Gastrisus*).

27. Mesothorax, mesocoxae: (0) Mesocoxae contiguous, intercoxal area distinctly recessed compared to mesoventrital and metaventrital processes (Fig. S158); (1) Mesocoxae moderately separated, intercoxal area distinctly recessed compared to mesoventrital process only (Fig. S87); (2) Mesocoxae strongly separated, intercoxal area on approximately same plane as both meso and metaventrital processes (Fig. S117).

28. Mesothorax, mesoscutellum, dense micropunctures: (0) absent (Fig. 2D); (1) present (Fig. 2C).

29. Mesoventrite, intercoxal process, apex: (0) narrow and pointed (Fig. S60); (1) broad and rounded; (2) narrow and rounded (Fig. 2E); (3) broad and pointed (Fig. 2F).

30*. Metathorax, metepisternum, punctures: (0) dorsal 1/3 of metepisternum without punctures throughout its length (Fig. 3A); (1) metepisternum covered with punctures or impunctate area less than 1/3 (Fig. 3B).

31*. Metathorax, relative width of metepimeron in comparison to metepisternum near posterior border: (0) metepimeron subequal or slightly wider than metepisternum (Fig. 3A); (1) metepimeron twice as wide as metepisternum (Fig. 3B).

32*. Metathorax, metacoxae, spines on the posterior surface: (0) 4 or less (Fig. 3C); (1) more than 4 (Fig. 3D). This character is difficult to observe and sometimes spines may have been broken off.

33*. Metathorax, metafemora, upper posterior margin: (0) crenulate (Fig. 3G); (1) not crenulate.

**Figure 4.**
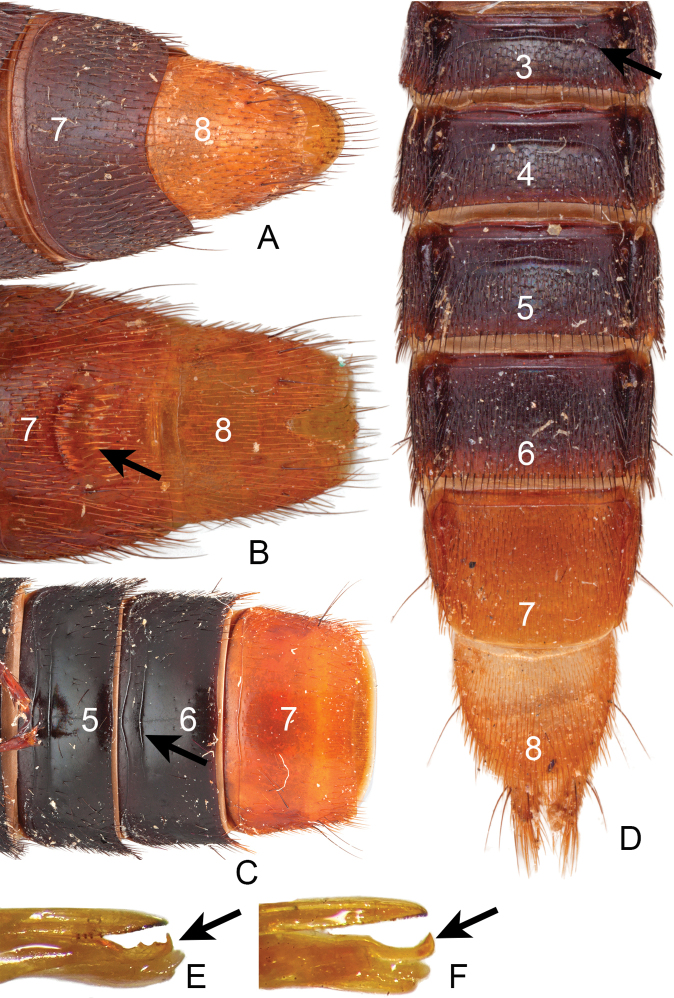
Diagnostic characters for *Xanthopygus***A** abdominal sternites 7–8 of *Xanthopygusskalitzkyi* (Bernhauer) **B** abdominal sternites 7–8 of *Xanthopygusviridipennis* Sharp, arrow points to the porose structure **C** abdominal sternites 5–7 of *Xanthopygusgiganeus* (Bernhauer), arrow points to the anterior transverse lines **D** abdominal tergites 3–8 of *Xanthopyguscognatus* Sharp, arrow points to arch-like carina on tergite 3 **E** lateral view of the aedeagus of *Xanthopygusfaustus* (Erichson), arrow points to the serrated apical carina **F** lateral view of the aedeagus of *Xanthopygusdimidiatus* Bernhauer, arrow points to the hook-like carina. Not to scale.

34. Metathorax, metatarsi, tarsomere 3, dorsal surface, chaetotaxy: (0) developed only at margins, dorsal surface of tarsomeres glabrous (or with 1–2 setae) along midline (Fig. 3E); (1) tarsomeres dorsally setose (setae not restricted to marginal series) (Fig. 3F).

35. Abdomen, tergites 3 and 4, anterior basal transverse carina (ABTC), pair of accessory ridges: (0) absent (Fig. 4D); (1) present (Fig. CA1–9).

36. Abdomen, tergite 3, curved carina (arch-like) on disc: (0) absent; (1) present (Fig. 4D).

37. Abdomen, tergite 3, punctation medially: (0) absent; (1) present (Fig. 4D).

38. Abdomen, tergite 5, curved carina (arch-like) on disc (if curved carina present on tergite 3): (0) absent; (1) present (Fig. 4D).

39. Abdomen, sternite 3, basal transverse carina, medial area: (0) straight to arcuate (Fig. L18C); (1) acutely pointed medially (Fig. L18A, D).

40. Abdomen, sternite 5, dense, meshed microsculpture anterolaterally, appearing different in texture to posterior portion (microsculpture more obvious than normal punctures): (0) absent; (1) present (Fig. CH23–34).

41*. Abdomen, sternite 6, two anterior transverse lines: (0) absent; (1) present (Fig. 4C).

42. Abdomen, sternite 7, punctation laterally (excluding micropunctures): (0) sparse (each row of punctures separated by more than two puncture width from other rows) (Fig. 4A); (1) dense (punctures contiguous or rows separated by less than two puncture width) (Fig. 4B).

43. Male, abdomen, sternite 7, emargination of posterior margin (in comparison to female sternite 7): (0) absent; (1) present (Fig. 4A–C).

44. Male, abdomen, sternite 7, degree of emargination of posterior margin if present: (0) broad and shallow (Fig. 4B–C); (1) narrower and more pronounced (Fig. 4A).

45. Male, abdomen, sternite 7, porose structure: (0) absent (Fig. 4A, C); (1) present (Fig. 4B).

46. Male, abdomen, sternite 7, shape of porose structure (if present): (0) circular and pit-like, typically with few modified setae (Fig. CA19); (1) broad and brush-like, with many modified setae (Fig. 4B).

47. Male, abdomen, sternite 8, emargination: (0) shallow (just a notch) (Fig. 4A); (1) U-shaped; (2) deep U-shaped (1/3–1/4 length of segment) (Fig. 4B).

48. Male, aedeagus, median lobe, apical tooth: (0) absent; (1) present (Fig. CT5).

49. Male, aedeagus, tip of median lobe in dorsal view: (0) pointed (Fig. CA53); (1) rounded (Fig. CA112); (2) broadly expanded (Fig. CA71).

50*. Male, aedeagus, median lobe, serrated apical carina: (0) absent; (1) present (Fig. 4E).

51*. Male, aedeagus, median lobe, hook-like carina: (0) absent; (1) present (Fig. 4F).

### Phylogenetic analysis

Bayesian analysis were conducted in MrBayes v.3.2.7 (Ronquist et al. 2012) running on the CIPRES Science Gateway v3.3 (https://www.phylo.org). Convergence was assessed by examining the Potential Scale Reduction Factor (PSRF) and Average Standard Deviation of Split Frequency values (ASDSF) in the MrBayes output. The matrix (Suppl. material 2) was treated as a single partition and the analyses were performed using the Mkv model with gamma distribution and correction for ascertainment bias, with two runs of four chains each, default temperature (temp = 0.1) and 10,000,000 generations. I used the ‘trace all characters’ analysis in Mesquite to map all character states on the tree and the results of this analysis are presented as Suppl. material 3. A maximum parsimony analysis was not performed since Bayesian analysis outperforms parsimony for analysis of discrete morphological data (e.g., Wright and Hillis 2014; O’Reilly et al. 2016).

## Results

### Phylogenetic analysis

**Figure 5.**
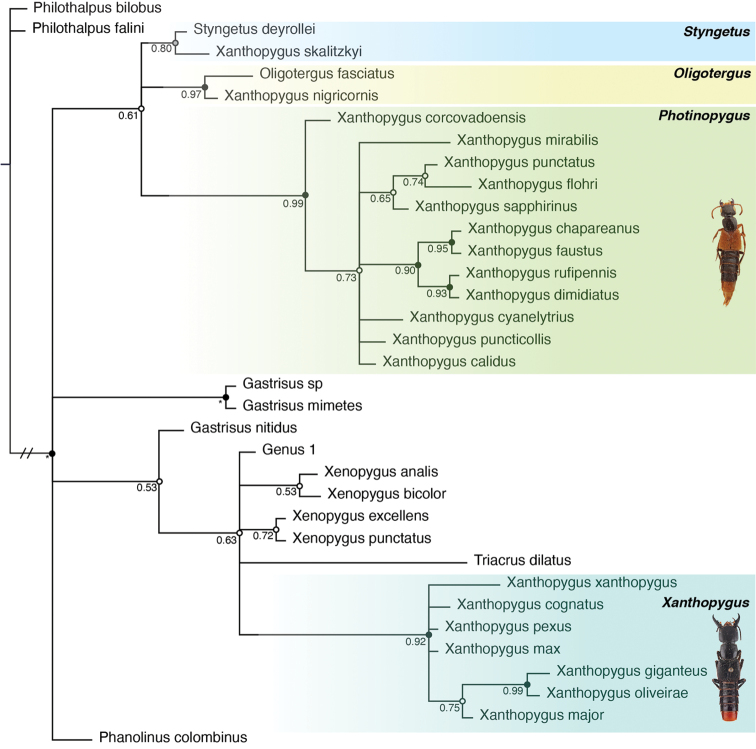
Fifty per cent majority rules consensus tree from a Bayesian phylogenetic analysis of 51 morphological characters. Posterior probabilities (PP) are given to the left of the corresponding node. Nodes colored based on support: PP ≥ 0.90 black; PP 0.80–0.89 grey; PP < 0.80 white.

The Bayesian analysis (Fig. 5) of the morphological matrix converged after 10 million generations with ASDSF = 0.001 and all PSRF values = 1.000. The analysis strongly supported the monophyly of the Xanthopygina (PP = 1) but most of the backbone clades were either weakly supported or not supported. Species from *Xanthopygus* sensu Herman appeared in four different parts of the phylogenetic tree (see below for details), and based on these results, *Xa.skalitzkyi* is transferred to *Styngetus* as *Styngetusskalitzkyi* comb. nov., *Xa.nigricornis* is transferred to *Oligotergus* as *Oligotergusnigricornis* comb. nov., a large group of *Xanthopygus* species are transferred to a new genus, named here *Photinopygus* gen. nov. (see Table 2 for details on the taxonomy) and the remaining taxa are left in *Xanthopygus* sensu nov.

**Table 1.** List of *Xanthopygus* species sensu Herman and their current name based on this paper. Bold type font on the first column indicates taxa included in the phylogenetic analysis. Taxa not included in this analysis but transferred to *Photinopygus* have all the diagnostic features of *Photinopygus*. Similarly, taxa that remained in *Xanthopygus* but were not included in the analysis have all the diagnostic features of *Xanthopygus* sensu novo.

Name sensu Herman 2001	Current status
*Xanthopygusalienus* Bernhauer, 1905	*Photinopygusalienus* (Bernhauer, 1905); comb. nov.
*Xanthopygusapicalis* Sharp, 1876	*Photinopygusapicalis* (Sharp, 1876); comb. nov.
*Xanthopygusborealis* Hatch, 1957	junior synonym of *Tympanophoruspuncticollis* (Erichson, 1840); (Moore & Legner 1975)
*Xanthopyguscacti* Horn, 1968	junior synonym of *Xanthopygusxanthopygus* (Nordmann, 1837); (Newton et al. 2000)
***Xanthopyguscalidus*** (Erichson, 1839)	*Photinopyguscalidus* (Erichson, 1839); comb. nov.
***Xanthopyguschapareanus*** Scheerpeltz, 1969	*Photinopyguschapareanus* (Scheerpeltz, 1969); comb. nov.
*Xanthopyguschrysopygus* (Nordmann, 1837)	*Photinopyguschrysopygus* (Nordmann, 1837); comb. nov.
*Xanthopyguschrysurus* (Nordmann, 1837)	*Photinopyguschrysurus* (Nordmann, 1837); comb. nov.
***Xanthopyguscognatus*** Sharp, 1876	*Xanthopyguscognatus* Sharp, 1876
*Xanthopyguscollaris* Bernhauer, 1925	*Photinopyguscollaris* (Bernhauer, 1925); comb. nov.
***Xanthopyguscorcovadoensis*** Scheerpeltz, 1969	*Photinopyguscorcovadoensis* (Scheerpeltz, 1969); comb. nov.
***Xanthopyguscyanelytrius*** (Perty, 1830)	*Photinopyguscyanelytrius* (Perty, 1830); comb. nov.
*Xanthopyguscyanipennis* Sharp, 1876	*Photinopyguscyanipennis* (Sharp, 1876); comb. nov.
*Xanthopygusdepressus* Sharp, 1876	*Photinopygusdepressus* (Sharp, 1876); comb. nov.
***Xanthopygusdimidiatus*** Bernhauer, 1917	*Photinopygusdimidiatus* (Bernhauer, 1917); comb. nov.
*Xanthopyguselegans* Bernhauer, 1905	*Photinopyguselegans* (Bernhauer, 1905); comb. nov.
***Xanthopygusfaustus*** (Erichson, 1839)	*Photinopygusfaustus* (Erichson, 1839); comb. nov.
***Xanthopygusflohri*** Sharp, 1884	*Photinopygusflohri* (Sharp, 1884); comb. nov.
*Xanthopygusgiganteus* (Bernhauer, 1906)	*Xanthopygusgiganteus* (Bernhauer, 1906)
*Xanthopygusgrimmeri*^a^ Duvivier, 1883	*nomen dubium*; (Herman 2001)
*Xanthopygushaemorrhoidalis* (Germar, 1824)	*Photinopygushaemorrhoidalis* (German, 1823); comb. nov.
*Xanthopygushilaris* (Erichson, 1839)	*Photinopygushilaris* (Erichson, 1839); comb. nov.
*Xanthopygusiopterus* (Erichson, 1939)	*Photinopygusiopterus* (Erichson, 1939); comb. nov.
*Xanthopygusjanthinipennis* (Blanchard, 1842)	*Photinopygusjanthinipennis* (Blanchard, 1842); comb. nov.
***Xanthopygusluctuosus*** (Blanchard, 1842)	*Xanthopygusluctuosus* (Blanchard, 1842)
***Xanthopygusmajor*** (Bernhauer, 1917)	*Xanthopygusmajor* (Bernhauer, 1917)
***Xanthopygusmax*** Blackwelder, 1944	*Xanthopygusmax* Blackwelder, 1944
***Xanthopygusmirabilis*** (Erichson, 1840)	*Photinopygusmirabilis* (Erichson, 1840); comb. nov.
*Xanthopygusmorosus* Sharp, 1884	*Photinopygusmorosus* (Sharp, 1884); comb. nov.
***Xanthopygusnigricornis*** Scheerpeltz, 1969	*Oligotergusnigricornis* (Scheerpeltz, 1969); comb. nov.
*Xanthopygusnigripes* Sharp, 1876	*Photinopygusnigripes* (Sharp, 1876); comb. nov.
***Xanthopygusoliveirae*** Lynch, 1884	*Xanthopygusoliveirae* Lynch, 1884
***Xanthopyguspexus*** (Motschulsky, 1858)	*Xanthopyguspexus* (Motschulsky, 1858)
***Xanthopyguspunctatus*** Bernhauer, 1905	*Photinopyguspunctatus* (Bernhauer, 1905); comb. nov.
***Xanthopyguspuncticollis*** Sharp, 1884	*Photinopyguspuncticollis* (Sharp, 1884); comb. nov.
***Xanthopygusrufipennis*** Sharp, 1884	*Photinopygusrufipennis* (Sharp, 1884); comb. nov.
***Xanthopygussapphirinus*** (Erichson, 1839)	*Photinopygussapphirinus* (Erichson, 1839); comb. nov.
***Xanthopygusskalitzkyi*** (Bernhauer, 1906)	*Styngetusskalitzkyi* (Bernhauer, 1906); comb. nov.
*Xanthopygustepidus* (Erichson, 1839)	*Photinopygustepidus* (Erichson, 1839); comb. nov.
*Xanthopygusviolaceipennis* Bernhauer, 1927	*Photinopygusviolaceipennis* (Bernhauer, 1927); comb. nov.
*Xanthopygusviolaceus* Sharp, 1876	*Photinopygusviolaceus* (Sharp, 1876); comb. nov.
*Xanthopygusviridipennis* Sharp, 1876	*Photinopygusviridipennis* (Sharp, 1876); comb. nov.
***Xanthopygusxanthopygus*** (Nordmann, 1837)	*Xanthopygusxanthopygus* (Nordmann, 1837)

^a^: The species was listed as *nomen dubium* by Herman (2001), and was originally described as distributed in Austria, which is peculiar given that no Xanthopygina are known from the Palearctic. I have contacted the Curator of Coleoptera in the Natural History Museum of Graz, Austria, where the Grimmer collection is housed and no taxa matching this name exist in the collection (Hausl-Hofstätter personal communication). It is unlikely that any specimens exist that can be attached to this name.

In a tree rooted by *Philothalpus*, all other taxa were placed in four different clades in a polytomy. The first clade contained *Phanolinuscolombinus*, and the second clade is composed of the sister groups *Gastrisus* sp. and *Gastrisusmimetes* (PP = 1). The third clade was unsupported (called here the *Xanthopygus* clade); it contained several species (*Gastrisusnitidus*, *Triacrusdilatus*, Genus 1 and the fours species of *Xenopygus*) and a large portion of the *Xanthopygus* species. The species of *Xanthopygus* in this clade formed a monophyletic group that was strongly supported (PP = 0.92) and will be treated as the *Xanthopygus* sensu nov. (for details see below on the Taxonomy section). Taxa included here were the ones placed in the genus *Lampropygus* by early taxonomists. *Xanthopygusgiganteus* was the sister group of *Xa.oliveirae* (PP = 0.99) and together were the sister group of *Xa.major* but without support. This clade was placed in a polytomy with *Xa.xanthopygus*, *Xa.cognatus*, *Xa.pexus* and *Xa.max*. For a list of characters that support Xanthopygus sensu nov. see the Taxonomy section below and Table 2.

**Table 2.** List of taxonomic characters that distinguish species of *Xanthopygus* from *Photinopygus*. Numbers next to characters refers to the numbers in the data matrix. For a full list of characters and character states see Material and Methods, and for the mapping of the characters on the phylogenetic tree see Suppl. material 3.

Characters	*Photinopygus*	*Xanthopygus*
4. Antennae, antennomere 5	(0) elongate (Figs 1A, F).	(1) subquadrate (Figs 1D–E).
8. Head, width in comparison to pronotum	(1) subequal (Figs 1A–C).	(2) wider^1^ (Figs 1D–E) (apomorphy).
9. Head, shape, posterior margin	(0) slightly extended posteriad on each side of the neck^2^ (Figs 1A–C) (apomorphy).	(1) more or less at same level with neck border (Figs 1D–E).
10. Head, eye size relative to length of head	(1) medium (between 2/5 and 2/3 length of head) (Fig. 1B).	(0) small (less than 2/5 length of head) (Fig. 1D) (apomorphy).
15. Mandible, left, teeth structure	(2) one bicuspid tooth (Fig. 1C).	(1) two teeth, separated by deep emargination (Fig. 1D) (apomorphy).
17. Pronotum, microsculpture	(3) with sparse micropunctures (but no transverse lines visible at 70× magnification) (apomorphy).	(1) with transverse lines (seen easily at 70× magnification)^3^.
22. Prothorax, hypomeron, angles of superior and inferior marginal lines	(0) superior and inferior line produce anterolateral angles parallel to one other (Fig. 2A).	(1) superior and inferior line produce anterolateral angles not parallel to one other (Fig. 2B) (apomorphy).
28. Mesothorax, mesoscutellum, dense micropunctures	(0) absent (Fig. 2D) (apomorphy).	(1) present (Fig. 2C).
29. Mesoventrite, intercoxal process, apex	(2) narrow and rounded (Fig. 2E) (apomorphy).	(1) broad and rounded; or (3) broad and pointed (Fig. 2F).
30. Metathorax, metepisternum, punctures	(1) metepisternum covered with punctures or impunctate area less than 1/3^4^ (Fig. 3B).	(0) dorsal 1/3 of metepisterstum without punctures throughout its length (Fig. 3A) (apomorphy).
32. Metathorax, metacoxae, spines on the posterior surface	(0) 4 or less (Fig. 3C).	(1) more than 4^5^ (Fig. 3D).
34. Metathorax, metatarsi, tarsomere 3, dorsal surface, chaetotaxy	(1) tarsomeres dorsally setose (setae not restricted to marginal series) (Fig. 3F) (apomorphy).	(0) developed only at margins, dorsal surface of tarsomeres glabrous (or with 1–2 setae) along midline (Fig. 3E).

^1^ It should be noted that head size is sexually dimorphic in *Xanthopygus* (but always wider than pronotum) and head size can vary drastically among specimens of the same species similarly to what has been observed in *Smilax* (Chatzimanolis 2016) and *Triacrusdilatus* (Chatzimanolis 2015a; Marlowe et al. 2015); ^2^ Except *Ph.mirabilis* and *Ph.corcovadoensis* (9:1); ^3^ Except *Xa.giganteus* (17:0); ^4^ Except *Ph.mirabilis* (30:0); ^5^ Except *Xa.xanthopygus* (32:0).

The fourth clade (called here the *Photinopygus* clade) included *Xanthopygus* taxa in three different subclades. *Xanthopygusskalitzkyi* was placed as the sister group of *Styngetusdeyrollei* (Solsky) with weak support (PP = 0.80) and supported by a unique synapomorphy present in all *Styngetus* species: (character 33:0 and matrices in Suppl. material 2, 3) upper posterior margin of metafemur crenulate. *Xanthopygusnigricornis* was placed as the sister group of *Oligotergusfasciatus* (Nordmann) with strong support (PP = 0.97) and two unique (for Xanthopygina) synapomorphies (1:0) antennomere 1 less than twice as long as antennomere 2; and (15:0) left mandible with a single tooth (character state also present in *Philothalpus*).

The remaining taxa in the fourth clade all belonged in *Xanthopygus* sensu Herman and were strongly supported as a monophyletic group (PP = 0.99). *Xanthopyguspunctatus* was recovered as the sister group of *Xa.flohri* but without support (PP = 0.74) and together as the sister group of *Xa.sapphirinus* (PP = 0.65). That clade was placed in a polytomy with *Xa.mirabilis*, *Xa.cyanelytrius*, *Xa.puncticollis*, *Xa.calidus*, and a strongly supported clade (PP = 0.90) of *Xa.chapareanus* + *Xa.faustus* (PP = 0.95) as the sister group of *Xa.rufipennis* + *Xa.dimidiatus* (PP = 0.93). All these taxa previously in *Xanthopygus* are transferred to a new genus, *Photinopygus* gen. nov. and the apomorphies supporting this new genus are given below in the Taxonomy section and in Table 2.

### Taxonomy

#### Oligotergus

Taxon classificationAnimaliaColeopteraStaphylinidae

Bierig, 1937

F13DB80C-678A-59DD-A992-D19488F418E1

##### Type species.

Philothalpus (Oligotergus) oculatus, fixed by monotypy (Herman 2001).

##### Species included.

The genus includes 20 species listed in Newton (2021) and *Oligotergusnigricornis* comb. nov. based on the results of the phylogenetic analysis presented in this paper. For a complete taxonomic history of the genus see Herman (2001).

##### Diagnosis.

The genus is not revised so the following characters (in combination) should be considered only as a partial list: left mandible with single tooth; antennomere 1 less than twice as long as antennomere 2; eyes large; pronotum with dense micropunctures (not in all species).

##### Remarks.

The type species was not available for the phylogenetic analysis. A formal revision of the genus is forthcoming (Chatzimanolis in preparation) where all species belonging to this genus will be treated and illustrated.

#### Styngetus

Taxon classificationAnimaliaColeopteraStaphylinidae

Sharp, 1884

447E54C0-1462-504D-B377-18635469CE87

Fig. 1F

##### Type species.

*Philonthusviduus* Erichson, fixed by subsequent designation by Blackwelder (1952) (Herman 2001).

##### Species included.

The genus includes 16 species listed in Newton (2021) and *Styngetusskalitzkyi* comb. nov. based on the results of the phylogenetic analysis presented in this paper. For a complete taxonomic history of the genus see Herman (2001).

##### Diagnosis.

The genus is not revised so the following characters (in combination) should be considered only as a partial list: left mandible with bicuspid tooth; protarsi without ventral pale macrosetae (not present in all taxa); metafemur with upper posterior margin crenulate; sternites 3–5 with arch-like carina.

##### Remarks.

The type species was not available for the phylogenetic analysis. A formal revision of the genus is forthcoming (Chatzimanolis in preparation) where all species belonging to this genus will be treated and illustrated.

#### Photinopygus

Taxon classificationAnimaliaColeopteraStaphylinidae

Chatzimanolis gen. nov.

57F63C0A-54AD-5592-A856-D8C2AC1A5D57

http://zoobank.org/ab8578bb-db63-4f34-a863-109d68a05bb9

Figs 1A–C

##### Type species.

*Staphylinuscalidus* Erichson, here designated.

##### Species included.

*alienus*, *apicalis*, *calidus*, *chapareanus*, *chrysopygus*, *chrysurus*, *corcovadoensis*, *cyanelytrius*, *cyanipennis*, *depressus*, *dimidiatus*, *elegans*, *faustus*, *flohri*, *haemorrhoidalis*, *hilaris*, *iopterus*, *janthinipennis*, *mirabilis*, *morosus*, *nigripes*, *punctatus*, *puncticollis*, *sapphirinus*, *tepidus*, *violaceipennis*, *violaceus* and *viridipennis* (see Table 1 for complete names).

##### Diagnosis.

This genus can be distinguished from all other genera in Xanthopygina based on the combination of the following characteristics: head shape rectangular; posterior margin of head slightly extended posteriad on each side of the neck (apomorphy; except in *Ph.corcovadoensis* and *Ph.mirabilis*); antennomeres 1–5 elongate; labial palpomere 3 not securiform; medium size eyes; superior marginal line of pronotal hypomeron not continuing to anterior margin; postcoxal process present; pronotum with sparse micropunctures but no transverse lines visible at 70× magnification (apomorphy); mesoscutellum without dense micropunctures (apomorphy); mesoventral process narrow and rounded (apomorphy); metatarsi with setose dorsal surface (apomorphy); tergite 3 (at minimum, some species 3–4 or 3–5) with arch-like carina; and sternite 7 in males with emargination at posterior margin. For a list of characters that distinguish *Photinopygus* from *Xanthopygus*, see Table 2.

##### Etymology.

The name is a combination of the Greek words φωτεινός (shining, bright) and πυγή (rump), and refers to the bright coloration of abdominal segments 7 and 8. The name is masculine.

##### Remarks.

A formal revision of the genus is forthcoming (Chatzimanolis in preparation) where all species belonging to this genus will be treated and illustrated. Even though some of the species transferred to *Photinopygus* were not included in the phylogenetic analysis, they can be confidently placed in this genus since they have all the diagnostic features of *Photinopygus* (see Tables 1 and 2 for details).

#### Xanthopygus

Taxon classificationAnimaliaColeopteraStaphylinidae

Kraatz, 1857 sensu novo

2D0E21CA-99C2-5FF0-9D7F-F1DBEAD0D93F

Figs 1D–E

##### Type species.

*Staphylinusxanthopygus* Nordmann, 1837, fixed by absolute tautonymy (Herman 2001).

##### Species included.

*cognatus*, *giganteus*, *luctuosus*, *major*, *max*, *oliveirae*, *pexus* and *xanthopygus*. (see Table 1 for complete names and Herman 2001 for taxonomic history).

##### Diagnosis.

This genus can be distinguished from all other genera in Xanthopygina based on the combination of the following characteristics: head shape rectangular; head wider than pronotum (apomorphy; however, head size can be variable among specimens of the same species but wider than pronotum); antennomeres 7–10 transverse; left mandible with two teeth separated by deep emargination (apomorphy); labial palpomere 3 not securiform; small size eyes (apomorphy); superior marginal line of pronotal hypomeron not continuing to anterior margin; superior and inferior marginal line of hypomeron produce anterolateral angles not parallel to one other (apomorphy); postcoxal process present; elytra coloration black (except with blue metallic overtones in *Xa.xanthopygus*); dorsal 1/3 of metepisterstum without punctures (apomorphy; state also present in *Ph.mirabilis*); with more than four spines on the posterior surface of metacoxae (apomorphy; less than four in *Xa.xanthopygus*); tergites 3–5 with arch-like carina; and sternite 7 in males with emargination at posterior margin. For a list of characters that distinguish *Xanthopygus* from *Photinopygus*, see Table 2.

##### Remarks.

A formal revision of the genus is forthcoming (Chatzimanolis in preparation) where all species belonging to this genus will be treated and illustrated.

## Discussion

The phylogenetic analysis presented here strongly rejected the hypothesis that *Xanthopygus* sensu Herman is a monophyletic group. As was previously defined, *Xanthopygus* included species that belonged in four distinct (and, as far as it is known, they are not sister to each other) clades, the genera *Oligotergus*, *Photinopygus*, *Styngetus* and *Xanthopygus*. The classification changes implemented in this paper resolve this issue by defining *Xanthopygus* in a new sense that includes some species that were described in *Lampropygus* (a synonym of *Xanthopygus*), although of the four species originally included in *Lampropygus* (Sharp 1884) two are now placed in *Xenopygus* (*Xe.analis* and *Xe.bicolor*, both included in the analysis here). However, *Lampropygus* was never clearly defined and included species (e.g., *L.skalitzkyi*) that clearly did not belong in that genus. Most of the species that belonged in *Xanthopygus* sensu Herman are placed in the new genus *Photinopygus*. Both *Xanthopygus* sensu novo and *Photinopygus* as presented in this paper are well-defined with clear diagnostic features that would hopefully prevent future misplaced species in these genera.

*Styngetusskalitzkyi* and *Oligotergusnigricornis* were clearly placed in *Xanthopygus* sensu Herman by mistake by Bernhauer (1906) and Scheerpeltz (1969), respectively. In both of these species, the superior marginal line of the hypomeron continues to the anterior end, which should have been a clear indication that the placement in *Xanthopygus* sensu Herman was erroneous. Granted, both of these species are atypical for either *Styngetus* or *Oligotergus* and these genera are still in dire need of revision since they contain multiple species of uncertain affinities (Chatzimanolis, unpublished data), not to mention the lack of clearly defined diagnostic features. Most species of *Styngetus* have a much narrower head than *Styngetusskalitzkyi* and some species of *Styngetus* have narrow protarsi (not seen in *Styngetusskalitzkyi*). However, the crenulate upper posterior margin of the metafemur is present in all species of *Styngetus* examined by me (and *Styngetusskalitzkyi*) and seems to be a good diagnostic character for the genus, pending its further review and phylogenetic analysis. In any case, *Styngetus* is probably more homogeneous than *Oligotergus* as currently defined. *Oligotergus* seems to include at least two distinct species groups, roughly split into species with dense small uniform punctures on the pronotum and species with larger, less dense punctures on pronotum. *Oligotergusnigricornis* belongs in the second group and whether these two species groups both should be included in *Oligotergus* is matter of further investigation.

One of the major issues with *Xanthopygus* sensu Herman was that the characters used to define the genus (superior marginal line of pronotal hypomeron not continuing to anterior margin, postcoxal process present, and tergites 3–5 with arch-like carina) are not unique to *Xanthopygus* and the genus was not easily recognizable. Even if somebody were to argue that the phylogeny presented here is not properly resolved, meaning that perhaps *Xanthopygus* sensu novo and *Photinopygus* might be sister groups and therefore do not have to be in separate genera, the reality is that *Xanthopygus* sensu Herman was impossible to diagnose with just the characters presented above. Perhaps more importantly, *Photinopygus* and *Xanthopygus* sensu novo do not share any apomorphies that could be used to diagnose *Xanthopygus* sensu Herman 2001. The split of *Xanthopygus* sensu Herman into *Xanthopygus* sensu novo and *Photinopygus* makes both of these genera easily recognizable and identifiable, given the characters presented in Table 2.

One of the characters used to define *Xanthopygus* sensu Herman was the superior marginal line of pronotal hypomeron not continuing to anterior margin. Until recently, it was not clear how widespread this character state is among Xanthopygina. *Triacrusdilatus* has the same character state and this feature along with the position of *Triacrus* on the phylogeny of Xanthopygina (Chatzimanolis and Brunke 2019) led these authors to hypothesize that perhaps *Triacrus* belonged in *Xanthopygus* (but see below for characters that exclude *Triacrus* from *Xanthopygus*). However, the particular state of the superior marginal line of pronotal hypomeron seen in *Xanthopygus* and *Triacrus* is more common than previously thought and certain species of *Gastrisus*, Genus 1, and even some species of *Plociopterus* Kraatz exhibit this particular character state. It is likely that this character state is much more widespread in Xanthopygina than previously documented and has evolved multiple times in several different lineages. Additionally, given the parallel evolution, this character state alone should not be used as a justification for a hypothetical close relationship between *Xanthopygus* sensu novo and *Photinopygus*.

A caveat in this paper is that the backbone relationships presented are unsupported. This is certainly not uncommon in morphology-only analyses using Bayesian statistics, and previous morphology-only Bayesian analyses of Staphylinini had low support values (e.g., Chatzimanolis and Brunke 2019, 2021). Usually combining morphological with molecular data alleviates nodes with low posterior probability values. But one problem with the addition of molecular data in papers that target relationships among species within genera rather than among higher taxonomic groups is the scarcity of DNA-grade material. For example, several of the species of *Xanthopygus* sensu novo are only known from the type and/or few additional specimens. While modern techniques have enabled the use of museum specimens in molecular analyses of Staphylininae (e.g., Brunke et al. 2021), using type materials for DNA analyses is still a sensitive subject with museum curators. Also, such techniques are expensive and thus may not be feasible for smaller standalone projects like this paper. Even though molecular data would have improved the resolution of the relationships presented here, the goal of this paper was to eliminate an obvious non-monophyletic group, *Xanthopygus* sensu Herman. For this purpose, the morphology-only analysis presented here was adequate and clearly indicated that *Xanthopygus* sensu Herman was polyphyletic. Discovering the exact phylogenetic placement of every species is a pending future task. The phylogenetic analysis presented in this paper differs to the one presented by Chatzimanolis and Brunke (2019, 2021) regarding the placement of *Photinopygus* among Xanthopygina lineages. The analysis presented in this paper indicated *Photinopygus* belonging in a different lineage of Xanthopygina (the *Plociopterus* lineage) than *Xanthopygus* (that belongs in the *Xanthopygus* lineage) but that result was unsupported (in terms of posterior probabilities). However, further analyses are needed to test how closely related *Photinopygus* and *Xanthopygus* may be.

The phylogenetic position of *Xenopygus* within the *Xanthopygus* lineage of genera remains unresolved. Chatzimanolis and Caron (2016) proposed two species groups within *Xenopygus* (*punctatus*, which includes *Xe.punctatus* and *Xe.excellens*, and *analis*, which includes *Xe.analis* and *Xe.bicolor*) and cautioned that these species groups may need to be placed in different genera in the future. In Chatzimanolis and Brunke (2019) the two species of *Xenopygus* (*Xe.excellens* and *Xe.analis*) included did not form a monophyletic group. In this paper, I added two more species (*Xe.punctatus* and *Xe.bicolor*) in the analysis, hoping that the addition of these taxa may help clarify their phylogenetic position. The analysis in this paper failed to find support for a monophyletic *Xenopygus* and it is unclear if morphological data alone can resolve this puzzle. In any case, it seems unlikely (and unsupported by the current data) that the *analis* species group of *Xenopygus* and *Xanthopygus sensu novo* are closely related as had been hypothesized early on by Sharp (1884) by his placement of these taxa in *Lampropygus*.

Likewise, the position of *Triacrus* remains unresolved. In Chatzimanolis and Brunke (2019)*Tr.dilatus* was placed as the sister group of *Xa.chapareanus* (now *Photinopyguschapareanus*) and in Chatzimanolis and Brunke (2021) in a polytomy with *Xa.xanthopygus* and *Xa.chapareanus*. In this paper, *Tr.dilatus* is in a polytomy with Genus 1, *Xenopygus* and the clade that leads to *Xanthopygus* sensu novo. While the exact position of *Tr.dilatus* is unclear, it is likely that this species is not closely related to *Photinopygus*, and current data does not support its placement within *Xanthopygus* sensu novo. *Triacrus* can easily be excluded from *Photinopygus* or *Xanthopygus* sensu novo based on the shape of antennomere 5 (transverse), the shape of teeth on left mandible (one large tooth and one bicuspid tooth) and the lack of postcoxal process.

It is perhaps unfortunate that most of the species that used to belong in *Xanthopygus* sensu Herman required a new name and were transferred to *Photinopygus*. However, this action corrected existing taxonomic problems and was necessary. Unfortunately, changing the meaning of an existing genus name is not uncommon; for example, Chatzimanolis and Ashe (2005) completely changed the meaning of *Philothalpus*, and multiple other times a genus name has been drastically redefined in Xanthopygina (e.g., *Dysanellus* Bernhauer: Chatzimanolis 2012; *Trigonopselaphus* Gemminger and Harold: Chatzimanolis 2015b; *Torobus* Herman: Chatzimanolis 2018). It is very likely that further changes in the name usage might be necessary in Xanthopygina as revisionary work progresses, especially in poorly defined genera such as *Gastrisus* or *Oligotergus*.

## Conclusions

The Bayesian phylogenetic analysis performed in this paper showed that *Xanthopygus* sensu Herman is polyphyletic. To solve this problem, one species was transferred to *Oligotergus*, another to *Styngetus*, a new genus *Photinopygus* was erected for many taxa previously in *Xanthopygus* and *Xanthopygus* sensu novo was restricted to the remaining species. The new diagnostic characters provided in this paper can be easily used to define *Photinopygus* or *Xanthopygus*. Even though this paper helped to untangle the relationships within *Xanthopygus* sensu Herman, the exact relationships of the genera within the *Xanthopygus* lineage are still uncertain and would probably require comprehensive molecular phylogenetic analyses to decipher.

## Supplementary Material

XML Treatment for Oligotergus

XML Treatment for Styngetus

XML Treatment for Photinopygus

XML Treatment for Xanthopygus

## Supplementary materials

Supplementary material 1 DarwinCore Data type Occurences. Explanation note DarwinCore format with all types and additional materials examined for the phylogenetic analysis. https://binary.pensoft.net/file/612034This dataset is made available under the Open Database License (http://opendatacommons.org/licenses/odbl/1.0/). The Open Database License (ODbL) is a license agreement intended to allow users to freely share, modify, and use this Dataset while maintaining this same freedom for others, provided that the original source and author(s) are credited.Stylianos Chatzimanolis

Supplementary material 2 Matrix Data type Data matrix. Explanation note The data matrix in .nex format. https://binary.pensoft.net/file/612035This dataset is made available under the Open Database License (http://opendatacommons.org/licenses/odbl/1.0/). The Open Database License (ODbL) is a license agreement intended to allow users to freely share, modify, and use this Dataset while maintaining this same freedom for others, provided that the original source and author(s) are credited.Stylianos Chatzimanolis

Supplementary material 3 Trace over trees Data type Analysis. Explanation note The analysis of “trace all characters” in Mesquite presented in .nex format. https://binary.pensoft.net/file/612036This dataset is made available under the Open Database License (http://opendatacommons.org/licenses/odbl/1.0/). The Open Database License (ODbL) is a license agreement intended to allow users to freely share, modify, and use this Dataset while maintaining this same freedom for others, provided that the original source and author(s) are credited.Stylianos Chatzimanolis

## References

[B1] Bernhauer M (1905) Neue Staphyliniden aus Südamerika. Deutsche Entomologische Zeitschrift 1905: 177–187. 10.1002/mmnd.48119050305

[B2] Bernhauer M (1906) Neue Staphyliniden aus Südamerika. Deutsche Entomologische Zeitschrift 1906: 193–202. 10.1002/mmnd.48019060105

[B3] Bernhauer M (1917) Neue südamerikanische Staphyliniden. Wiener Entomologische Zeitung 36: 102–116. 10.5962/bhl.part.12925

[B4] Bernhauer M (1927) Beitrag zur Staphylindenfauna Südamerikas insbesondere Braziliens. Memorie della Società Entomologica Italiana 5(2)(1926): 152–169

[B5] Blackwelder RE (1943) Monograph of the West Indian beetles of the family Staphylinidae. United States National Museum Bulletin 182: 1–658. 10.5479/si.03629236.182.i

[B6] Blackwelder RE (1952) The generic names of the beetle family Staphylinidae, with an essay on genotypy. United States National Museum Bulletin 200: 1–483.

[B7] Brunke AJ Hansen AK Salnitska M Kypke JL Predeus AV Escalona H Chapados JT Eyres J Richter R Smetana A Ślipiński A Zwick A Hájek J Leschen RA Solodovnikov A Dettman JR (2021) The limits of Quediini at last (Staphylinidae: Staphylininae): a rove beetle mega-radiation resolved by comprehensive sampling and anchored phylogenomics. Systematic Entomology 46: 396–421. 10.1111/syen.12468

[B8] Caron E de Castro JC Da Silva MR Ribeiro-Costa CS (2016) Phylogeny and revision of a colorful Neotropical genus of rove beetles: *Xenopygus* Bernhauer (Coleoptera: Staphylinidae). Zootaxa 4138(1): 59–82. 10.11646/zootaxa.4138.1.2 27470752

[B9] Chani‐Posse MR Brunke AJ Chatzimanolis S Schillhammer H Solodovnikov A (2018) Phylogeny of the hyper‐diverse Philonthina rove beetles with implications for classification of the tribe Staphylinini (Coleoptera: Staphylinidae). Cladistics 38: 1–40. 10.1111/cla.12188

[B10] Chatzimanolis S (2012) *Zackfalinus*, a new genus of Xanthopygina (Coleoptera: Staphylinidae:Staphylinini) with description of 20 new species. Annals of the Carnegie Museum 80(4): 261–308. 10.2992/007.080.0401

[B12] Chatzimanolis S (2014) Phylogeny of xanthopygine rove beetles based on six molecular loci. Systematic Entomology 39(1): 141–149. 10.1111/syen.12040

[B13] Chatzimanolis S (2015a) New records, redescription, and notes on nomenclature for *Triacrus* Nordmann (Coleoptera: Staphylinidae: Staphylininae: Staphylinini). The Coleopterists Bulletin 69(3): 514–520. 10.1649/0010-065X-69.3.514

[B14] Chatzimanolis S (2015b) A revision of the genus *Trigonopselaphus* Gemminger and Harold (Coleoptera: Staphylinidae: Staphylininae). Koleopterologische Rundschau 85: 167–189.

[B15] Chatzimanolis S (2016) A revision of the myrmecophilous genus *Smilax* Laporte (Coleoptera: Staphylinidae: Staphylininae). Zootaxa 4162(2): 283–303. 10.11646/zootaxa.4162.2.5 27615974

[B16] Chatzimanolis S (2017) And then there were six: a revision of the genus *Phanolinopsis* Scheerpeltz (Coleoptera: Staphylinidae: Staphylininae). Zootaxa 4323(1): 49–67. 10.11646/zootaxa.4323.1.4

[B17] Chatzimanolis S (2018) A review of the genera *Dysanellus* Bernhauer and *Torobus* Herman (Staphylinidae: Staphylininae: Staphylinini). The Coleopterists Bulletin 72(2): 279–291. 10.1649/0010-065X-72.2.279

[B18] Chatzimanolis S (2019) *Lendatus*, a new genus of Xanthopygina (Coleoptera: Staphylinidae: Staphylininae) with description of three new species. PeerJ 7: e7947. 10.7717/peerj.7947 PMC6815202 31660278

[B19] Chatzimanolis S Ashe JS (2005) Revision and phylogeny of the neotropical genus *Philothalpus* (=*Eugastus* Sharp and *Allostenopsis* Bernhauer) (Coleoptera: Staphylinidae: Xanthopygina). Insect Systematics and Evolution 36: 63–119. 10.1163/187631205788912813

[B20] Chatzimanolis S Brunke AJ (2019) A phylogeny of Xanthopygina (Insecta, Coleoptera) reveals major lineages and the origin of myrmecophily. Zoologica Scripta 48(4): 494–506. 10.1111/zsc.12358

[B21] Chatzimanolis S Brunke AJ (2021) A new apterous rove beetle genus (Coleoptera: Staphylinidae) from the Northern Andes with an assessment of its phylogenetic position. European Journal of Taxonomy 744: 67–82. 10.5852/ejt.2021.744.1303

[B22] Chatzimanolis S Caron E (2016) New species and synonymies in *Xenopygus* Bernhauer (Staphylinidae: Staphylinini). Zootaxa 4200(1): 131–142. 10.11646/zootaxa.4200.1.5 27988642

[B23] Chatzimanolis S Hightower HJ (2019) *Peripus*, a new genus of Xanthopygina (Coleoptera: Staphylinidae) from South America. Zootaxa 4648(2): 371–383. 10.11646/zootaxa.4648.2.10 31716954

[B24] Erichson WF (1839) Genera et species Staphylinorum insectorum coleopterorum familiae. Berlin: FH. Morin, 1–400. 10.5962/bhl.title.59644

[B25] Erichson WF (1840) Genera et species Staphylinorum insectorum coleopterorum familiae. Berlin: FH. Morin, 401–954. 10.5962/bhl.title.59644

[B26] Gemminger M von Harold E (1868) Catalogus Coleopterorum hucusque descriptorum synonymicus et systematicus. Vol. III. Monachii: Sumptu Gummi EH. 10.5962/bhl.title.9089

[B27] Hayashi Y (1997) Studies on the Asian Staphylininae (Coleoptera, Staphylinidae). III. The characteristics of the Xanthopygini. Elytra 25: 475–492.

[B28] Herman LH (2001) Catalog of the Staphylinidae (Insecta: Coleoptera). 1758 to the end of the second millennium. Parts I‐VII. Bulletin of the American Museum of Natural History 265: 1–4218. 10.1206/0003-0090.265.1.1

[B29] International Commission on Zoological Nomenclature (1999) International Code of Zoological Nomenclature, 4^th^ edn., adopted by the International Union of Biological Sciences. London: International Trust for Zoological Nomenclature.

[B30] Kraatz G (1857) Naturgeschichte der Insecten Deutschlands. Abt. 1. Coleoptera. Zweiter Band. Berlin: Nicolai, Lief. 3–4 pp. 377–768, Lief. 5–6 pp. 769–1080.

[B31] Lucas R (1920) Catalogus alphabeticus generum et subgenerum Coleopterorum orbis terrarum totius (famil., trib., subtr., sect. incl.). Archiv für Naturgeschichte (A) 84(1918): 1–696.

[B32] Li L Zhou H-Z (2011) Revision and phylogenetic assessment of the rove beetle genus *Eccoptolonthus* Hayashi, with broad reference to the subtribe Philonthina (Coleoptera: Staphylinidae: Staphylinini). Zoological Journal of the Linnean Society 163: 679–722. 10.1111/j.1096-3642.2011.00731.x

[B33] Maddison WP Maddison DR (2019) Mesquite: a modular system for evolutionary analysis. Version 3.61 http://www.mesquiteproject.org

[B34] Marlowe MH Murphy CA Chatzimanolis S (2015) Sexual dimorphism and allometry in the sphecophilous rove beetle *Triacrusdilatus*. PeerJ 3: e1123. 10.7717/peerj.1123 PMC4525698 26246969

[B35] Moore I Legner EF (1975) A catalogue of the Staphylinidae of America North of Mexico (Coleoptera). University of California Division of Agricultural Sciences Special Publication 3015: 1–514.

[B36] Navarrete-Heredia JL (2004) Sinopsis del género *Xanthopygus* Kraatz, 1857 (Coleoptera: Staphylinidae) de México. Acta zoológica mexicana 20(3): 1–13.

[B37] Navarrete-Heredia JL Newton AF Thayer MK Ashe JS Chandler DS (2002) Guía ilustrada para los géneros de Staphylinidae (Coleoptera) de México. Mexico: Universidad de Guadalajara and CONABIO.

[B38] Newton AF Thayer MK Ashe JS Chandler DS (2000[2001]) Staphylinidae Latreille, 1802. In: Arnett Jr. RH, Thomas MC (Eds) American Beetles. Archostemata, Myxophaga, Adephaga, Polyphaga: Staphyliniformia, vol. 1. CRC Press, Boca Raton, 272–418.

[B39] Newton AF (2021) StaphBase: Staphyliniformia world catalog database (version Nov 2018). In: Catalogue of Life, et al. 2021. Species 2000 and ITIS Catalogue of Life, 2021-04-05. Digital resource at www.catalogueoflife.org. Species 2000: Naturalis, Leiden, the Netherlands. ISSN 2405–8858.

[B40] O’Reilly JE Puttick MN Parry L Tanner AR. Tarver JE. Fleming J Pisani D Donoghue PCJ (2016) Bayesian methods outperform parsimony but at the expense of precision in the estimation of phylogeny from discrete morphological data. Biology Letters 12(4): e20160081. 10.1098/rsbl.2016.0081 PMC4881353 27095266

[B41] Quezada JR Amaya CA Herman LH (1969) *Xanthopyguscognatus* Sharp (Coleoptera: Staphylinidae), an enemy of the coconut weevil, *Rhynchophoruspalmarum* L. (Coleoptera: Curculionidae) in El Salvador. Journal of the New York Entomological Society 20: 264–269.

[B42] Rodríguez DT García GDA Navarrete-Heredia JL (2012) Sinopsis de los géneros de Xanthopygina (Coleoptera: Staphylinidae: Staphylinini) en Colombia. Dugesiana 18(2): 217–241.

[B43] Ronquist F Teslenko M van der Mark P Ayres D Darling A Höhna S Larget B Liu L. Suchard M Huelsenbeck J (2012) MrBayes 3.2: efficient Bayesian phylogenetic inference and model choice across a large model space. Systematic Biology 61(3): 539–542. 10.1093/sysbio/sys029 22357727 PMC3329765

[B44] Scheerpeltz O (1969) Die zentral- und südamerikanischen Arten der Gattung *Xanthopygus* Kraatz. (Col. Staphylinidae, Subfam. Staphylininae, Tribus Xanthopygini). Koleopterologische Rundschau 46/47: 109–118.

[B45] Sharp D (1876) Contribution to an insect fauna of the Amazon Valley (Col. Staph.). Transactions of the Entomological Society of London 1876: 27–424. 10.5962/bhl.title.5536

[B46] Sharp D (1884) Staphylinidae. In Godman FD, Salvin O (Eds). Biologia Centrali‐Americana, insecta, coleoptera, Vol. 1(2). London: Taylor and Francis, 145–392.

[B47] Smetana A Davies A (2000) Reclassification of the north temperate taxa associated with Staphylinus sensu lato, including comments on relevant subtribes of Staphylinini (Coleoptera: Staphylinidae). American Museum Novitates 3287: 1–88. 10.1206/0003-0082(2000)287<0001:ROTNTT>2.0.CO;2

[B48] Wright AM Hillis DM (2014) Bayesian analysis using a simple likelihood model outperforms parsimony for estimation of phylogeny from discrete morphological data. PLoS ONE 9(10): e109210. 10.1371/journal.pone.0109210 PMC4184849 25279853

